# Tag SNP Polymorphism of *CCL2* and Its Role in Clinical Tuberculosis in Han Chinese Pediatric Population

**DOI:** 10.1371/journal.pone.0014652

**Published:** 2011-02-04

**Authors:** Wei-Xing Feng, Igor Mokrousov, Bin-Bin Wang, Hugh Nelson, Wei-Wei Jiao, Jing Wang, Lin Sun, Si-Rui Zhou, Jing Xiao, Yi Gu, Xi-Rong Wu, Xu Ma, Adong Shen

**Affiliations:** 1 Beijing Pediatric Research Institute, Beijing Children's Hospital affiliated to Capital Medical University, Beijing, China; 2 Laboratory of Molecular Microbiology, St Petersburg Pasteur Institute, St Petersburg, Russia; 3 Graduate School, Peking Union Medical College, Beijing, China; 4 National Research Institute for Family Planning, Beijing, China; 5 International SOS Clinic, Shekou, Shenzhen, China; Institut de Pharmacologie et de Biologie Structurale, France

## Abstract

**Background:**

Chemokine (C-C motif) ligand 2 CCL2/MCP-1 is among the key signaling molecules of innate immunity; in particular, it is involved in recruitment of mononuclear and other cells in response to infection, including tuberculosis (TB) and is essential for granuloma formation.

**Methodology/Principal Findings:**

We identified a tag SNP for the *CCL2*/*MCP-1* gene (rs4586 C/T). In order to understand whether this SNP may serve to evaluate the contribution of the *CCL2* gene to the expression of TB disease, we further analysed distribution of its alleles and genotypes in 301 TB cases versus 338 non-infected controls (all BCG vaccinated) representing a high-risk pediatric population of North China. In the male TB subgroup, the C allele was identified in a higher rate (*P* = 0.045), and, acting dominantly, was found to be a risk factor for clinical TB (*P* = 0.029). Homozygous TT genotype was significantly associated with lower CSF mononuclear leukocyte (ML) counts in patients with tuberculous meningitis (TBM) (*P* = 0.001).

**Conclusions/Significance:**

The present study found an association of the *CCL2* tag SNP rs4586 C allele and pediatric TB disease in males, suggesting that gender may affect the susceptibility to TB even in children. The association of homozygous TT genotype with decreased CSF mononuclear leukocyte (ML) count not only suggests a clinical significance of this SNP, but indicates its potential to assist in the clinical assessment of suspected TBM, where delay is critical and diagnosis is difficult.

## Introduction

With 9 million new cases of tuberculosis (TB) worldwide in 2008 [1], the disease caused by *Mycobacterium tuberculosis* remains a topmost health problem in China with over 1.3 million new cases/year and a prevalence as high as 194/100,000 in adults and 98/100,000 in children [1], [2]. Although more than one-third of the world's population is potentially infected with *M. tuberculosis*, only 10% will develop the clinical disease over a lifetime [3]. Some genetic factors that may account for the development of TB disease after infection include (list not exhaustive): chemokine(C-C motif) ligand 2 (CCL2, other designation: MCP-1), genes encoding human leukocyte antigen type, purinergic receptor (P2X_7_), the solute carrier family 11 member a1 protein (SLC11A1, formerly known as NRAMP1), and vitamin D3 receptor [4], [5], [6], [7], [8], [9], [10] although the data may vary among different ethnic groups.

CCL2 belongs to the CC chemokines characterized by two adjacent cysteine residues close to the amino terminus. As with many other CC chemokines, the *CCL2* gene is located on chromosome 17q11.2 in humans which has been linked to susceptibility to tuberculosis and includes genes coding for several chemokines where *CCL2* clusters together with the nitric oxide synthase 2A (*NOS2A*) and *CCL3-5* genes [11], [12]. The CCL2 chemokine is produced as a protein precursor containing signal peptide of 23 amino acids and a mature peptide of 76 amino acids [13], [14]. The chemokine CCL2 is the most potent chemo-attractant and activator for mononuclear leukocytes (MLs) and attracts CD4 and γδ T cells [15], [16], [17], which are central components of the granulomatous response [18]. CCL2 can be released directly from infected cells and by forming a chemical gradient, escort sensitive MLs to the site of tissue injury [19]. Expression of the *CCL2* gene may be found in many disorders characterized by ML infiltration [20]. CCL2 (MCP-1) plasma levels were significantly higher in TB adult patients of Mexican and Han Chinese ancestry who were carriers of -2518 *CCL2* genotype GG [4], [7]. CCL2 binds to CCR2 receptors and was shown to down-regulate the production of IL-12 and selectively inhibit the induction of the Th1 immune response to *Staphylococcus aureus* [21], [22]. The association of -2518G allele with TB was found in North-East Asian (north China and Korea) [4], [5], [7], Zambian [23] and Central-South American (Mexico, Peru) populations [7], [8] whereas lack of such an association was found for South-East Asian (South China/Hong Kong) [18], South African Colored and Indian [24], [25] populations. In contrast, the *CCL2* -2518G allele was significantly associated with resistance to TB in Ghanaians [26].

Childhood TB represents an important and sensitive part of the burden of TB in the community. Compared to adults, children are much more likely to progress to TB disease after exposure, to develop disease more rapidly, and to develop asymptomatic extra-pulmonary disease, disseminated (miliary) TB and tuberculous meningitis (TBM) [27], [28], [29], [30], [31]. It has been suggested that disseminated TB may reflect Mendelian predispositions in a fraction of children (3–30% estimated by Bayesian statistics), whereas adult pulmonary TB seems to reflect a more complex genetic predisposition [32], [33].

Unlike previous studies that focused on the *CCL2* promoter SNPs, we hereby report a tag SNP for the entire *CCL2* gene region. We further evaluated whether its polymorphism may serve to elucidate the contribution of the *CCL2* gene to the expression of TB disease in a BCG-vaccinated pediatric population in North China. We also compared the *CCL2* genotypes and counts of the MLs and polymorphonuclear (PMN) cells in the cerebrospinal fluid (CSF) in the TBM subgroup.

## Results

### TB patients and non-infected controls

The case group (n = 301) included 105 (34.9%) cases of active pulmonary TB (PTB), 196 (65.1%) cases of extrapulmonary TB (EPTB), of which was 118 (39.2%) cases of TBM. Severe extrapulmonary tuberculosis (SevTB) identified in 172 (57.1%) patients included those with two or more noncontiguous sites, miliary mottling, or involvement of meningitis, pericardium, spinal, intestinal and splenic sites with or without lung involvement. The LNTB (Less severe extrapulmonary tuberculosis) group included 24 (8%) patients with extra-pulmonary peripheral sites restricted to lymph nodes, peripheral joints, skin or pleural effusions without involvement of lung parenchyma. The control group included 338 pediatric surgical patients without history of tuberculosis. The demographic characteristics are explained in Table 1. All study subjects were BCG vaccinated at birth. The mean age was 5.9 years (SD, 4.8; range, 2 months–17 years) in the TB patients group. The mean age in control group (n = 338) was 5.7 years (SD, 4.1; range, 3 months–16 years). The proportions of male were observed 61.8% in both groups. There were no significant differences between the groups for age and sex. The children aged less than 4 years old constituted 48.6%, 46.5%, 16.7% in PTB, SevTB and LNTB groups, respectively.

**Table 1:** Demographic characteristics of study population.

Characteristic*	Number of TB patients*					Control (n = 338)
	PTB (n = 105)	SevTB (n = 172)	LNTB (n = 24)		Total (n = 301)	
Gender						
Male	68(64.8)	100(58.1)	18(75.0)	186(61.8)		209(61.8)
Age						
Mean (SD), Y	5.4 (4.7)	5.8(4.7)	9.5(4.7)	5.9(4.8)		5.7(4.1)
Age group, Y						
<4	51(48.6)	80(46.5)	4(16.7)	135(44.9)		131(38.7)
4–6	14(13.3)	21(12.2)	2(8.3)	37(12.3)		73(21.6)
7–9	18(17.1)	22(12.8)	5(20.8)	45(14.9)		58(17.2)
10–12	11(10.5)	33(19.2)	7(29.2)	51(16.9)		58(17.2)
13–17	11(10.5)	16(9.3)	6(25.0)	33(11.0)		18(5.3)
BCG Vaccinated	Yes	Yes	Yes	Yes		Yes

*PTB, pulmonary TB; SevTB, severe extrapulmonary TB; LNTB, Less severe extrapulmonary TB; TBM, TB meningitis.

All TB cases were classified according to the diagnostic standards of the American Thoracic Society (ATS) [34], the Pediatric tuberculosis clinical diagnosis standard in China [35] and WHO guidelines for disease severity classification for non-HIV related TB [36]. Patients were included in the study if they were positive by one or more of the following criteria: Tspot-TB test, Acid-Fast Bacilli Stain, culture, imaging, fibreoptic bronchoscopy (FOB) observation. The clinical records of patients were reviewed by two pediatricians with experience of TB in children (Table 2).

**Table 2:** Diagnostic modality used for confirmation of TB.

Investigation or diagnostic procedure	Number of TB patients (positive/total)*	
	PTB(n = 105)	EPTB(n = 196)
PPD	100/105	169/196
Tspot-TB test	21/21	31/31
Acid-Fast Bacilli Stain	8/105	12/196
Culture	8/105	22/196
Imaging	105/105	155/196
FOB Observation	25/35	16/20

*PTB, pulmonary TB; EPTB, extrapulmonary TB; FOB, fibreoptic bronchoscopy.

### Tagged-Haplotype and Linkage Disequilibrium (LD) analysis

**Figure 1:**
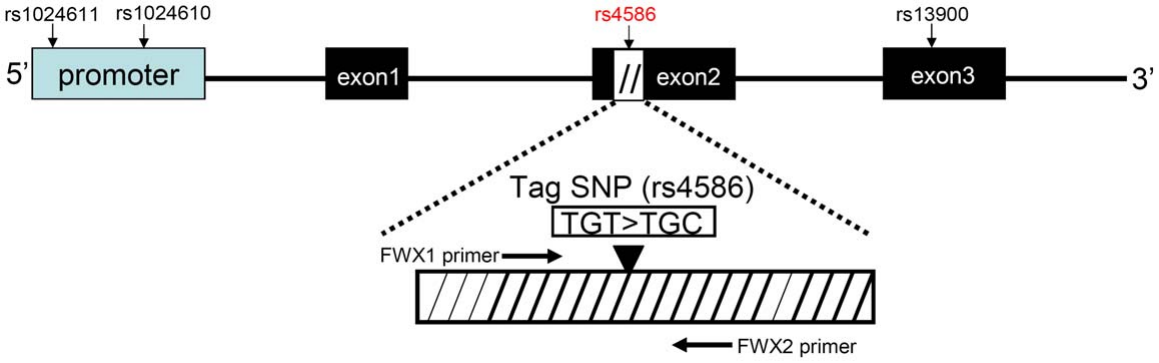
*CCL2* gene structure and location of tag SNP rs4586 (T/C), a synonymous mutation at codon 35-Cys in exon 2 of *CCL2*.

In the *CCL2* region (chr17:29603409..29609331, from 3000 bp 5′ upstream to 1000 bp 3′ downstream of *CCL2*)the Haploblock was constructed according to solid spine method and consisted of four SNPs (Fig. 1). These included two SNPs of promoter region rs1024611 (-2518A/G), rs1024610, one of exon 2 rs4586 and one of 3′UTR rs13900. Of this haplotype block, rs4586 was identified as the tag SNP for further association analysis. As the frequency of rs4586-C was similar to rs1024611-G, it was reasonable to expect that the rs4586-C and rs11024611-G (-2518G) alleles would be in perfect LD (D' = 0.975 R^2^ = 0.9, based on China Han Beijing CHB dataset in HapMap PHASE 3: http://hapmap.ncbi.nlm.nih.gov/).

### Analysis of rs4586 variants versus disease phenotype and gender

The genotypic and allelic frequencies of the tag SNP rs4586 of the *CCL2* were analyzed in cases and controls. The genotypes were in HWE (Hardy-Weinberg equilibrium). The frequency of the C allele in TB patients was 61.3%, whereas T allele frequency was 38.7%; these frequencies did not differ significantly between cases and controls (*P* = 0.576). Analysis of genotypic distribution using 3×2 χ^2^ test revealed no significant difference between the two groups (*P* = 0.664). Moreover, no significant difference in the genotypic or allelic frequencies was found: (i) when control group was compared to different patient subgroups stratified by diagnosis; (ii) between PTB and EPTB subgroups (iii) between SevTB and LNTB subgroups (Table 3).

**Table 3:** Effect of *CCL2* rs4586 variants on disease phenotype.

Subjects (n) *	Genotype, n (%)			*P*-value	Allele		*P*-value
	CC	TC	TT		C(%)	T(%)	
Control (338)	117(34.6)	170 (50.3)	51 (15.1)	-	404 (59.8)	272(40.2)	-
Case (301)	106 (35.2)	157 (52.2)	38 (12.6)	0.664	369(61.3)	233 (38.7)	0.576
PTB (105)	36 (34.3)	57 (54.3)	12 (11.4)	0.605	129 (61.4)	81(38.6)	0.667
EPTB (196)	70(35.7)	100 (51.0)	26 (13.3)	0.843	240 (60.6)	156 (39.4)	0.786
SevTB (172)	63(36.6)	85(49.4)	24(14.0)	0.883	211(61.3)	133(38.7)	0.627
LNTB (24)	7(29.1)	15(62.5)	2(8.0)	0.462	29(60.4)	19(39.6)	0.929
SevTB vs LNTB	-	-	-	0.464	-	-	0.902
PTB vs EPTB	-	-	-	0.834	-	-	0.843

*PTB, pulmonary tuberculosis; EPTB, extrapulmonary; SevTB, severe extrapulmonary tuberculosis; LNTB, Less severe extrapulmonary tuberculosis.

C is ancestral allele, T – derived allele when compared to the sequence of the chimpanzee.

No significant differences between the TB and control groups were observed among male and female in genotypic frequencies (data not shown). At the same time, it was noted that C allele was more frequent in the male TB group (2×2 test, *P* = 0.045; OR = 1.34; 95%CI 1.01–1.79). Different models of inheritance have been tested for combined sample and sex-stratified subsamples. In the male group, a statistical significance was observed when the C allele was regarded as a Mendelian dominant trait (*P* = 0.029; OR = 1.94; 95%CI 1.06–3.56). In the female group no statistically significant differences were found (Table 4).

**Table 4:** Relationship between *CCL2* rs4586 polymorphisms and pediatric tuberculosis (TB), stratified by sex.

Alleles and genotypes stratified by sex		TB	Control	χ^2^-value*	*P*-value	OR (95%CIl)
Male	C	243 (65.3)	244 (58.4)	4.02	0.045	1.34 (1.01–1.79)
	T	129(34.7)	174(41.6)			
Female	C	126 (54.8)	160 (62.0)	2.62	0.105	1.35 (0.94–1.93)
	T	104(45.2)	98(38.0)			
Male	CC+TC	168(90.3)	173(82.8)	4.75	0.029	1.94 (1.06–3.56)
	TT	18(9.7)	36(17.2)			
Female	CC+TC	95(82.7)	114(88.4)	1.64	0.200	0.63(0.30–1.29)
	TT	20(17.3)	15(11.6)			

*Genotype χ2 value for comparison of case and control groups in the genotype and allele frequencies.

In genetic studies the power of a sample set to detect an association depends on the allele frequency and the size of its effect (estimated as OR). For complex infectious diseases, e.g., tuberculosis, a mild effect of many contributing genes is more expected; therefore we adopted the OR of 1.5. Accordingly, the power to detect association for the minor allele T of the tag SNP in combined sample was found to be high (95%). Indeed under the stratification analysis, the power was lower (80% for males, 62% for females) and this was a limitation of this study. Considering the difficulties in recruiting children and the relatively smaller size of the total pediatric TB population (compared to adult TB), the design of our study may be considered appropriate. Indeed, similar case-control studies previously described have enrolled an even smaller number of individuals [37], [38], [39].

### Analysis of genotype versus CSF cells counts in TBM cases

**Figure 2:**
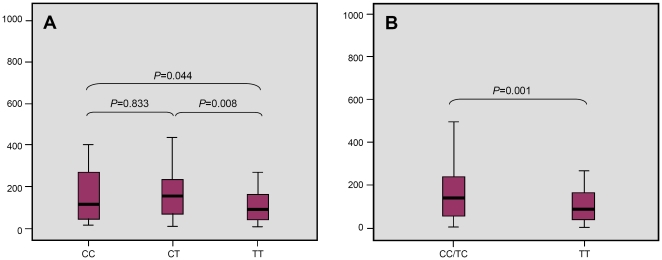
Numbers of MLs (e^6^/L) in CSF in different *CCL2* tag SNP rs4586 genotype groups in TBM patients. (**A**) Comparison of homo- and heterozygotes. (**B**) Testing dominant model for common allele C.

The data on ML and PMN counts in CSF were available for 110 of 118 TBM patients. The MLs count was decreased in TT genotype as compared with CC (*P* = 0.044) and TC (*P* = 0.008) genotypes. However, no difference was observed between CC and TC (*P* = 0.833) in the MLs level (Fig. 2A). The effect of the *CCL2* rs4586 minor allele T on the level of MLs in CSF was assessed using both dominant (CT+TT versus CC) and recessive (TT versus CC+CT) models. This comparison revealed a statistical significance for recessive model for T allele *(P* = 0.001) (Fig. 2B). The same analysis found no significant association of either genotype/allele and the number of PMNs in CSF (data not shown).

## Discussion

CCL2 is essential for the recruitment of mononuclear cells and subsequent activation of T lymphocytes, mononuclear and polymorphonuclear cells at the site of infection [17]. Studies of knockout mice indicated a non-redundant role of CCL2 in modulating MLs infiltration during inflammation [40]. Its involvement in pathogenesis of TB and granulomatous response suggested that variation of the *CCL2* gene might play a role in conferring susceptibility to or protection from TB. However, knockout-mice lacking *CCL2* were not found to be more susceptible to intravenous infection with *M. tuberculosis* [41] and published studies on the *CCL2* variation from different world regions produced somewhat controversial conclusions [7], [8], [26].

In this study, knowledge-based approach and linkage analysis using tagged-haplotype SNP were used in detecting *CCL2* genetic associations: we identified a single tag SNP for the entire *CCL2* gene (rs4586) and studied its role in clinical TB in Han Chinese pediatric population. Many studies demonstrated that haplotype diversity can be reduced to a smaller set of “haplotype-tagging” SNPs (htSNPs) identifying the most common haplotypes [42], [43]. Tag SNPs are useful in whole-genome SNP association studies in which hundreds of thousands of SNPs across the entire genome are genotyped since being representative SNPs in a genome region with high LD, they make it possible to identify genetic variation without genotyping every SNP in a chromosomal region of interest [44], [45]. Here, rs4586 was chosen as haplotype-tagging SNP in order to reduce genotyping requirements by eliminating redundancy in the information provided by the other SNPs in the extended *CCL2* genome region.

Our data indicated that a large proportion of cases were severe extrapulmonary TB (SevTB). No statistically significant differences were observed between cases of PTB and cases of SevTB. A comparison of carriers of the rs4586 genotype CC between SevTB and LNTB shows an increased proportion of homozygous CC among patients with SevTB (36.6% vs 29.1%, Table 3). Although this difference is not significant (OR 1.4, 95% CI 0.55–3.57), it provides an interesting clue. In Chinese Han population (Hapmap-CHB) rs4586-C and -2518-G are in perfect LD (D' = 0.975 *r* ^2^ = 0.9). Consequently, we speculate that the -2518 *CCL2* genotype GG may be associated with a more severe disease in a pediatric population of Han Chinese as well as in Koreans, Mexicans and Peruvians [4], [5], [7], [8] but not in non-Caucasian Africans, Indians and Caucasians [24], [25], [26]. Our analysis has a number of limitations (small sample size of the LNTB subgroup, difference in the mean age between SevTB and LNTB groups, 5.75 vs 9.45 years old, respectively) hence a larger study is needed to validate these preliminary results.

The significant associations were observed when results were stratified by gender. The CC and CT genotypes and the common C allele were significantly more prevalent in the male TB subgroup. As rs4586-C was found to be associated with susceptibility to TB in males, we speculate that -2518-G may also be associated with TB in Chinese male children. This hypothesis should be verified in the further study focused specifically on the -2518 SNP. The prevalence of TB varies on the basis of sex that may be a modifier in TB development [46], [47]. It is possible that a gender-related gene is involved in modification of TB development, and additional research must be performed to clarify the biological effects of gender in TB disease. However, as the *P*-value was at borderline significant level (0.045), a larger study will give better indications to the role of this polymorphism in susceptibility to TB in males.

**Figure 3:**
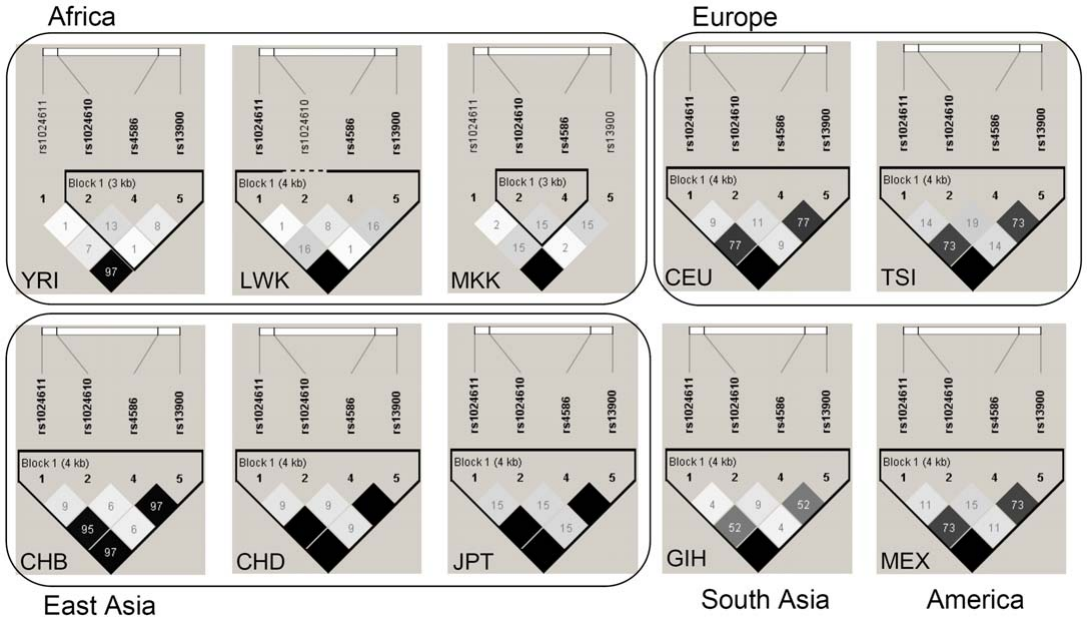
Pairwise LD (*r* ^2^) plots of *CCL2* variants in different populations based on preliminary data extracted from the HapMap Phase 3 release. YRI, Yoruban in Ibadan, Nigeria; LWK, Luhya in Webuye, Kenya; MKK, Maasai in Kinyawa, Kenya; CEU, the US residents of northern and western European ancestry; TSI, Tuscan in Italy; CHB, Han Chinese in Beijing, China; CHD, Chinese in Metropolitan Denver, Colorado; JPT, Japanese in Tokyo, Japan; GIH, Gujarati Indians in Houston, Texas; MEX Mexican ancestry in Los Angeles, California.

Unfortunately, no population data on rs4586 in relation to clinical TB are available yet for other populations whereas *CCL2* promoter SNP -2518 (rs1024611) has been extensively analyzed for its association with clinical TB. In this view, we extended our discussion to this latter SNP located inside the genome region under study and found to be in LD with rs4586 in our population. We would like to note that the association of -2518 with clinical TB observed in some but not all populations might reflect a genuine association owing to LD with another, putatively causal polymorphism in the adjacent *CCL2* gene region. When comparing the linkage plot of Chinese CHB population to the plots of the three African populations, clear differences of LD patterns became evident (Fig. 3). This is in general agreement with the known distinct LD patterns that exist in African and diverse non-African populations. East Asian (Chinese and Japanese) populations had very similar patterns of LD (for the whole haplotype block of 4 SNPs). In contrast, the association of *CCL2* rs4586-C was independent of that of -2518G in all African populations. A moderate LD was found in the Caucasian (US residents of European ancestry and Italians), Gujarati Indians, and Mexicans populations (Fig. 3). Accordingly, different LD patterns between -2518G and a causal unidentified variant adjacent to *CCL2* in different populations might explain the association with TB in some but not all populations. To sum up, the role of the *CCL2* -2518 (and likely rs4586) in TB disease may differ for different large settings, Africans versus East Asians versus Europeans.

It is worth mentioning that a strong association for -362C allele (rs2857656) with protection against TB was shown in the Ghanaian population [26], a finding in clear disagreement with our results since this SNP is located well within our study region. Following the above discussion on -2518 SNP in different populations, it is not unlikely that this discrepancy may also well be explained by variation in the LD patterns in East Asians and Africans. The rs4586 is a useful tag-SNP in Han Chinese and perhaps may be useful in certain other ethnic groups.

Compared to the previous studies of *CCL2* and human susceptibility to TB [4], [5], [7], [8], [25], [26], this study was distinctive in three ways. First, the targeted rs4586 was a *CCL2* tag SNP thus permitting us to assess the entire gene and surrounding regions (+3000 bp in 5′UTR and +1000 bp 3′UTR). Second, we focused on pediatric TB that is known to be clinically and pathologically different from adult TB [33]. Third, the subjects in the study were BCG vaccinated. The studies in Mexico and Peru [8] also focused on BCG-vaccinated adult individuals, and they found that the *CCL2* -2518 GG and *MMP-1*-1607 2G/2G increased the likelihood of progression to PTB. In their study all the controls were latently infected individuals with positive PPD test and did not develop TB disease for a period of more than 2 years after exposure. Differences in study populations (children in this study and adults in [8]) may explain these disparate results, particularly with respect to the criteria for selection of controls.

In this view, it is important to mention the limitations encountered during the design of the present study. A perfect study of susceptibility to TB should rely on the selection of the latently infected individuals as controls. Tuberculin Skin Test (TST) is useful to detect infected individuals for research purposes: Flores-Villanueva's group found that 99% of heavily exposed adults with a TST>15 mm are also positive to a QuantiFeron-TB test in tube [8]. However a use of TST in BCG-vaccinated children in a high-prevalence country, such as China, is more than challenging. In China, immediate chemoprophylaxis/treatment is provided to all PPD-positive children in spite of the fact that BCG-vaccination may confound the diagnosis of infection. Even if the truly infected controls have been identified for the research purposes in this project, they would be treated and treatment/chemoprophylaxis might modify the course of infection regardless of the genetic profiles of the study subjects. Assuming equal exposure of both groups and since our controls remained uninfected during 2 years, we assumed “non-infected” status of the controls. In this case, we compared allele and genotype frequencies in tuberculosis patients with those non-infected controls, allowing us to distinguish susceptibility to infection from non-infection. An additional limitation of our study was that we did not have some detailed information for comparison between patients and controls, such as children's family history, habitat, family income, parents' educational level, family size. However, there is no logical reason why these differences should cause a specific SNP distribution to change, especially when the standard statistical precautions of measuring HWE and LD on other available SNP data are observed. Summing up, we believe that our decision to use non-infected children as controls was justifiable.

TB meningitis most commonly occurs in children aged 0 – 4 years in the world areas with a high TB prevalence, such as China, and in adults in the areas with a low TB prevalence [48]. TB meningitis is the most severe complication of human *M. tuberculosis* infection causing death in 10% of cases with advanced disease and severe neurologic sequelae in 80% of survivors [49]. In this pediatric TB study, 39.2% (118/301) of cases were TBM patients, of which 110 cases were tested for MLs counts in CSF.

The appearance of MLs in the CSF is an important hallmark of TBM [50]. Chemokines and their receptors play a key role in directing the movement of MLs into the central nervous system (CNS) in pathological states [51]. CCL2 is essential for the recruitment of MLs hence the number of MLs in CSF may indeed be associated with *CCL2* genotype. The MCP-1–deficient mouse study also indicated that CCL2 alone was responsible for mononuclear cell infiltration in several inflammatory models in vivo [41]. This prompted us to compare genotype versus phenotype, i.e., frequencies of the genotypes and alleles of the *CCL2* tag SNP with the number of MLs (and PMNs) in the CSF. We found that subjects homozygous for T allele had decreased number of MLs in CSF (Fig. 2). Otherwise, the MLs level was higher than PMN level in CSF of TBM patients (P = 0.02). The lack of association between CCL2 rs4586 genotypes and the number of the PMNs in the CSF is readily explained by the chemokine CCL2 more specific activity for MLs rather than PMNs.

However the mode of action of rs4586 polymorphism on the role of CCL2 in migration and attraction of MLs is unknown. Synonymous variations, in particular, are assumed to be functionally neutral both in clinical diagnosis and when measuring evolutionary distances between species. However, recent results suggested that silent mutations can have an effect on subsequent protein structure and activity [52], [53]. Using the cystic fibrosis transmembrane conductance regulator (CFTR) exon 12 splicing as a model, it was demonstrated that ∼25% of synonymous variations result in exon skipping and, hence, in an inactive CFTR protein [52]. Furthermore, artificial combinations of CFTR exon 12 synonymous and non-synonymous substitutions were incompatible with normal RNA processing. The *CCL2* tag SNP rs4586 (Cys35Cys) targeted in this study is located in exon 2 and may indeed affect the protein function at transcription control by inducing aberrant skipping of the mutant exon.

*M. tuberculosis* can cross the blood brain barrier (BBB) as a free (extracellular) organism or via infected MLs/PMNs [54]. The clinical manifestation of CNS tuberculosis is primarily a consequence of the inflammation which develops in response to *M. tuberculosis* in the CNS. Obstruction of the CSF by inflammatory infiltrate leads to hydrocephalus, and vasculitis contributes to infarction, causing potentially irreparable neurological damage [54]. Inhibition of this inflammation may therefore help in preventing the sequelae of CNS TB. Corticosteroids such as dexamethasone which suppress the production of inflammatory cytokines and chemokines lead to better outcomes and are recommended as adjunctive treatment for patients with TB meningitis [55], [56]. Accordingly, patients with genotype TT with lower CSF MLs counts than genotypes CC/TC (*P* = 0.001) may cause less severe disease upon MLs infiltration. The TT genotype associated with lower level of MLs infiltration appears to act as a protection factor against stronger inflammation/more severe disease progression. If the TT genotype is associated with more efficient bacilli reduction in early stages of infection, then there may be evidence of decreased caseation in lungs, and possibly a lower incidence of inflammatory sequelae such as obstructive hydrocephalus after TBM, in TT homozygotes. Knowing that a patient has TT genotype may help in the correct clinical assessment of suspected TBM, by putting less reliance on an elevated ML count in the CSF, and greater reliance on other indicators.

An area for future study might be to see if the number of ventriculoperitonea (VP) shunting procedures to prevent hydrocephalus is decreased in patients homozygous for TT allele. Lumbar puncture remains essential in the investigation of suspected meningitis. In this view, although the clinical significance of this SNP remains to be established, in practical terms it may be useful for simple, non-invasive, genotypic prediction of MLs low counts in CSF in different disorders. The knowledge that a patient has TT genotype will assist the clinician to maintain a high index of suspicion for TBM even when CSF ML count is low.

### Conclusion

The present study found an association of the *CCL2* tag SNP rs4586-C allele and pediatric TB disease in males, suggesting that the gender may affect the susceptibility to TB even in children. This study indirectly demonstrated that CCL2 may modulate the migration of MLs thereby influencing disease progression. The derived T allele, acting recessively, and TT genotype are associated with decreased level of MLs in CSF of TBM patients. The rs4586 SNP may be useful as a simple, non-invasive, genotypic marker able to predict low counts of MLs in CSF and aid in appropriate clinical assessment of suspected TBM.

## Materials and Methods

### Ethics Statement

The clinical investigation *Tag SNP Polymorphism of CCL2 and its Role in Clinical Tuberculosis in Han Chinese Pediatric Population* has been conducted according to the principles expressed in the Declaration of Helsinki. For research involving human participants, informed consent has been obtained from the patients or the guardian of the patients. The research has been approved by the Ethics Committee of the Beijing Children's Hospital.

### Study sample

The data from 301 case patients and 338 control subjects were included in this study. All study individuals were of the Han Chinese ethnicity. All the cases and controls were vaccinated with BCG at birth, which was confirmed by the presence of a scar in the left shoulder and vaccination records in the clinical charts.

A total of 307 blood samples were obtained from pediatric patients with TB admitted at the Beijing Children's Hospital (affiliated with Capital Medical University; Beijing, China) between February 2005 and August 2010. Patients originated from Beijing city and surrounding provinces in North China. Children admitted consecutively to Beijing Children's hospital with clinical tuberculosis, a negative HIV test, were eligible to enter the study. In this study, primary immunodeficiency (PID) was ruled out by clinical history, examination, and the two tests for serum immunoglobulin levels (IgG, IgM and IgA) and lymphocyte subpopulations (CD3+ T-cells, CD56+ natural killer (NK) cells, CD19+ B-cells and also CD4+ and CD8+ T-cells measured by flow cytometry). Six patients were excluded due to PID. No patients born with a height and weight bellow the 50 percentile were included. None of patients had history of HIV infection, malnutrition, previous history of conditions affecting immune function, receipt of immunosuppressive therapy or other lung disease. In addition, all cases were new TB cases: none had a history of previous TB or had received previous treatment for TB.

Control group subjects were recruited from those admitted to the Beijing Children's Hospital between June 2005 and November 2007. Controls were matched with TB patients by age, sex, and ethnicity. The control group included 338 pediatric surgical patients without history of TB, with normal radiographic examination findings, and with PPD skin test results <5 mm. The control group children were without the infection or the history of any inflammatory, autoimmune or infectious diseases, including TB and HIV infection. However, as we are dealing with a pediatric group, the possibility of a control to evolve as a case in future cannot be ruled out. All controls were therefore seen or questioned again 2 years after their initial visit, and their non-infection status was confirmed (with PPD skin test results <5 mm). In the control groups, 50 randomly selected individuals were tested to determine whether they were infected with *M. tuberculosis* using Tspot-TB test (Oxford Immunotec, Abingdon, UK), and negative results were identified. A positive Tspot-TB test has a much higher sensitivity than skin testing as an indicator of active and latent *M. tuberculosis* infection [57], [58].

### DNA analysis and selection of haplotype-based tagging SNP

Blood samples from children with TB and controls were collected and stored at −20°C. Genomic DNA was extracted from peripheral blood leukocytes according to standard methods.

We aimed to identify a set of SNPs that efficiently tag all of the common allelic variants and thus likely to tag most of the unknown common haplotypes in CCL2. The tag SNP was selected based on ability to tag surrounding variants (chr17:29603409..29609331 ) in the Han Chinese panel (Beijing, China) of the International HapMap project, NCBI build B36 assembly HapMap phase III (http://www.hapmap.org). Tagger software included in Haploview software 4.1 [59] was used to select tag SNP. The region used in tag SNP selection included a region from 3,000 bp 5 ′upstream to 1000 bp 3 ′downstream of *CCL2* (Fig. 1). The synonymous TGC>TGT variation at codon 35-Cys (rs4586) was the only tag SNP in *CCL2* obtained by aggressive tagging a minor allele frequency (MAF) of 0.15, a pairwise correlation coefficient *r* ^2^ of 0.80. LD was calculated and graphically displayed with the Haploview 4.2 software.

The rs4586 and its surrounding regions of *CCL2* gene exon 2 were amplified with primers designed by Gene Tool V 1.0.0.1 (Launcher Program for Bio Tools Inc. Applications), sense primer FWX1 5′-CCCTGGTGCTGATCATCTGGA-3′, and antisense primer FWX2 5′-GGTGGAGAGTGATGTTGGGGTTC-3′(Fig. 1). The cycling conditions were as follows: initial denaturation at 94°C for 3 min; 35 cycles of 94°C for 30 s, 58.9°C for 30 s, and 72°C for 30 s; and final elongation at 72°C for 10 min. PCR products were directly sequenced using Big Dye Terminator 3.1 chemistry (Applied Biosystems Inc.) with the above primers and run on a 3730xl DNA analyzer (ABI).

### Determination of MLs and PMNs in CSF

The Five or more lumbar punctures were obtained from children with tuberculous meningitis (TBM). Mononuclear leukocytes (MLs) and polymorphonuclear (PMN) cells in CSF were counted in a hemocytometer under a 100× oil immersion objective. Mononuclear leukocytes (MLs) are white blood cells with a one-lobed nucleus; two common forms of ML are monocytes and lymphocytes. Neutrophils together with basophils and eosinophils form part of the polymorphonuclear cell family (PMNs). The numbers were expressed as cells per cubic millimeter in undiluted CSF; the final value used in our analysis was the maximum value of all repetitions.

### Genetic and statistic analysis

Statistical analysis was carried out using the Statistical Package for Social Sciences version 13.0 (SPSS, Chicago, IL, USA). Differences between non contiguous variables, genotype distribution and allele frequency were tested by chi-square analysis. The student's *t*-test was used to compare the numbers of the cells in the CSF between two genotypes, and ANOVA (Games-Howell test) was used to compare the numbers of cells in CSF for multiple comparisons. Significant differences were indicated by a *P* value <0.05. Power calculation was performed with Quanto 1.2.4 software (http://hydra.usc.edu/gxe). LDs were calculated and graphically displayed with the Haploview 4.2 software.
