# Correction: Tag SNP Polymorphism of *CCL2* and Its Role in Clinical Tuberculosis in Han Chinese Pediatric Population

**DOI:** 10.1371/annotation/675f01a0-0f6e-4eb5-8cb1-ee8c5b5cbfe3

**Published:** 2011-04-05

**Authors:** Wei-Xing Feng, Igor Mokrousov, Bin-Bin Wang, Hugh Nelson, Wei-Wei Jiao, Jing Wang, Lin Sun, Si-Rui Zhou, Jing Xiao, Yi Gu, Xi-Rong Wu, Xu Ma, Adong Shen

funding source is missing from the financial disclosure information. The authors would like to add the following sentence to the funding section: "This study was supported by the grants of National Natural Science Foundation of China (No. 30672258, No. 30872788 and No.30973981) and the grant from Beijing Municipal Science and Technology Commission (No.Z09050700940903)."

